# Wound Dehiscence after Wisdom Tooth Removal in Mandibular Mesioangular Class IB Impactions: Triangular Transposition Flap versus Envelope Flap

**DOI:** 10.15171/joddd.2015.032

**Published:** 2015-09-16

**Authors:** Amin Rahpeyma, Saeedeh Khajehahmadi, Sina Ilkhani

**Affiliations:** ^1^Associate Professor of Oral and Maxillofacial Surgery, Oral and Maxillofacial Diseases Research Center, School of Dentistry, Mashhad University of Medical Sciences, Mashhad, Iran; ^2^Assistant Professor of Oral and Maxillofacial Pathology, Dental Research Center, School of Dentistry, Mashhad University of Medical Sciences, Mashhad, Iran; ^3^Post-graduate Student of Oral and Maxillofacial Surgery, Oral and Maxillofacial Diseases Research Center, School of Dentistry, Mashhad University of Medical Sciences, Mashhad, Iran

**Keywords:** Dehiscence, envelope flap, triangular transposition flap, wisdom tooth

## Abstract

***Background and aims.*** Wound dehiscence after lower third molar surgery extends the postoperative treatment period and may cause long-standing pain. The aim of this study was to compare wound dehiscence after removal of wisdom teeth in the most prevalent mandibular impaction (mesioangular class IB) by two different soft tissue flap designs.

***Materials and methods.*** Partially-erupted mandibular third molars with mesioangular class IB impaction (Pell and Gregory classification) were selected. Split mouth technique was used to compare the two flap designs (envelope vs. triangular transposition flap—TTF). The patients were recalled one week and a month later and rechecked for dehiscence, infection, and dry socket formation.

***Results.*** There were no cases of infection in either group. However, three cases of dry socket in the envelope group and four in the TTF group were recorded. In the envelope group, dehiscence occurred in 43% of cases during the first week, with 67% of cases being a large dehiscence (diameters of more than 5 mm). Extra appointments (those requested by the patient exclusively related to the problem of the hole distal to the second molar) were scheduled in 10% of cases in the envelope group. In the TTF group, dehiscence occurred during the first week for the same impaction in 19% of cases with large dehiscence cases occurring in 65% of cases and extra appointment rate at 4.1%.

***Conclusion.*** According to theresults in the evaluated operation, TTF may prevent postoperative wound dehiscence more probably than the envelope flap.

## Introduction

Wisdom tooth removal is the most prevalent surgery carried out by the oral surgeons.^1^ Attention to surgical details such as flap design, bone removal, or tooth sectioning has an important role in the success of surgery. One common problem after removal of wisdom teeth, especially semi-impacted ones in the mandible, is formation of a hole distal to the second mandibular molar.^2^This hole usually entraps food and debris, and creates bad odor. The search for optimal surgical approach in removing third molars is highly important.^3^Flap design is one of the factors influencing the severity of postoperative complications such as pain, trismus, swelling, and wound dehiscence.^4^ Envelope flap with a distal releasing incision is the most common approach for lower third molar surgery and mesioangular impaction is the most prevalent type of impaction in the lower jaw.^5,6^ Wound dehiscence after lower third molar surgery extends the postoperative treatment and might give rise to long-standing pain.^7^

Previous studies on primary and secondary closure techniques have indicated that secondary approaches have better clinical success, but there are situations in which primary closure of the socket is advised to allow faster mucosal healing and greater promotion of bone regeneration.^8^Socket coverage for prevention of osteomyelitis after tooth extraction has been strongly recommended in patients taking intravenous bisphosphonates or receiving preoperative radiotherapy, and those suffering from osteopetrosis.^9-11^ These groups and some selected ordinary patients can benefit from primary closure of surgically removed wisdom teeth.

The experience of the authors with triangular transposition flap encouraged us to carry out a study to compare dehiscence after surgical removal of mandibular third molars with envelope flap that is the most prevalent soft tissue design in this respect. The aim of this study was to determine which flap (triangular transposition flap or envelope flap) has a lower chance of soft tissue dehiscence when a surgeon decides to close the extraction socket after surgical removal of mandibular partially-erupted third molars with mesioangular class IB impaction.

## Materials and Methods

Partially-erupted mandibular third molars with mesioangular class IB impaction (Pell and Gregory classification) were selected. Based on this classification, in class I there is sufficient space between the ramus and the distal part of the second molar for the accommodation of the mesiodistal diameter of the third molar; in class B the occlusal plane of the impacted tooth is between the occlusal plane and the cervical line of the second molar. In mesioangular impaction, the angle between the occlusal plane or the line parallel to it and the longitudinal axis of the impacted mandibular third molar is between 31° and 60°.^12^

A split mouth technique was used to compare the two flap designs. The reason for the removal of wisdom teeth was orthodontist’s request and prophylactic considerations. Smokers and patients with systemic diseases such as diabetes mellitus were not included in this study. The age range of the patients was 17-25 years. Based on inclusion criteria, the mandibular second molars were fully erupted without distal surface caries or periodontal diseases. The soft tissue coverage of the third molars was healthy. A total of 120 teeth (60 patients) were selected. Selection of the right or left side and the flap design was random. Envelope or triangular transposition flap (TTF), a modification of the triangular flap, was used on one side and after one month, the other design was applied on the opposite side. The patients were recalled one week and one month later and the operation field was checked for dehiscence, infection, and dry socket formation. Dehiscence was defined as “separation between buccal and lingual mucosa, after primary closure of the wound.” Dehiscence size was measured with a Vernier ruler. The largest diameter was recorded. A diameter less than 5 mm was called small dehiscence and greater than 5 mm was considered as a large dehiscence. One month later, the other side was operated with the other technique by the same surgeon. If the first surgical field did not heal properly, the second surgery was postponed. The surgery duration was recorded and if the difference was more than five minutes between the left and right sides in a patient, the case was excluded from the study.

After the second surgery, the patients were visited twice (one week and one month later). Patients who referred to the surgeon between these two appointments for dehiscence in mucosa distal to the second molar were recorded. Reasons for extra appointments other than the dehiscence, like bleeding, trismus and swelling, were not considered. Pain was considered only if it was apparently correlated with the distal hole.

### Surgical Technique

Local anesthesia was achieved with a cartridge of 1.8 mL of lidocaine, with 1:80000 epinephrine, for inferior alveolar nerve and long buccal nerves.

**Figure 1.**
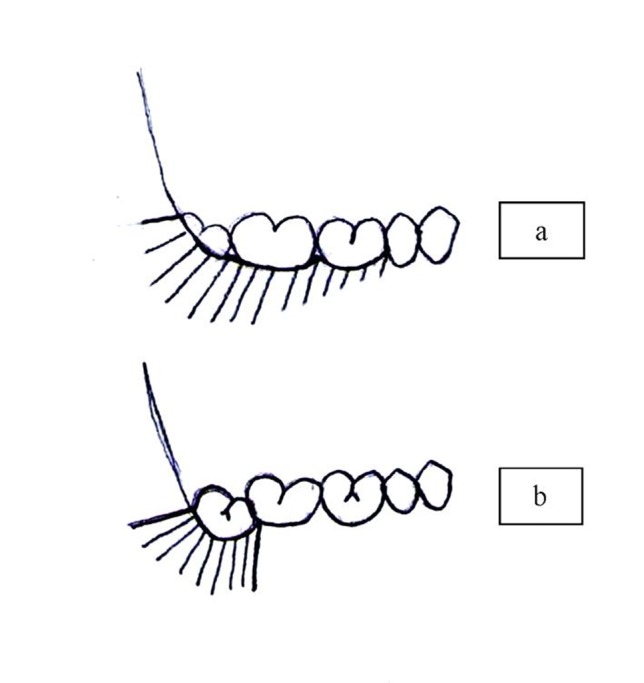
Schematic view of two flap design: (a) Envelope flap extends to the mesiobuccal line angle of first molar anteriorly. (b) Triangular transposition flap; the vertical release is placed at distobuccal line angle of second molar.

**Figure 2.**
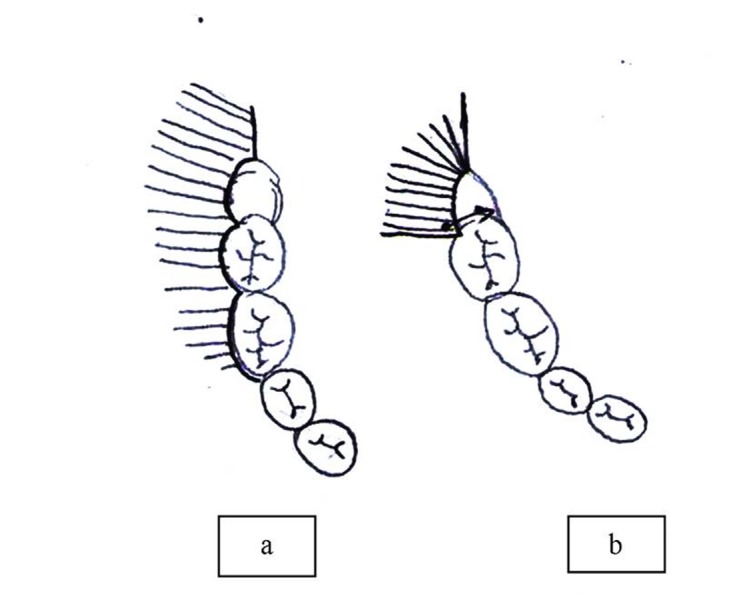
(a) First suture in envelope flap in distal surface of second molar. (b) Triangular transposition flap (TTF) transposed and sutured to the lingual gingiva adjacent to the distolingual line angle of the second molar.

Just before the beginning of surgical incisions another cartridge was injected subperiosteally to elevate the periosteum and assist in better elevation of the mucoperiosteal flap. In the envelope flap group, a sulcular incision was made around the neck of the first and second mandibular molars. The incision was extended into the sulcus of the semi-erupted third molar. The distal release was directed posteriorly (Figure 1a). After flap reflection, bone removal and tooth sectioning were carried out. After copious irrigation and rounding of the sharp edges of the socket wall, the envelope flap was sutured. The first suture was applied distal to the second molar and two other sutures were added anterior and posterior to the first one (Figure 2a). In the triangular transposition flap group (TTF), the vertical mesial releasing incision was placed back on the distobuccal line angle of the mandibular second molar, instead of the mesiobuccal line angle in routine triangular flaps, and extended vertically toward the vestibule at least one centimeter (Figure 1b).

The incision extended posteriorly in a manner similar to the envelop flap and the surgical procedure began and continued as above. At the end of the operation, during the suturing, the triangular flap was not sutured to the initial site but it was transposed to cover the extraction socket; it was sutured to the lingual gingiva, adjacent to the distolingual line angle of the second molar (Figure 2b). Two other sutures were applied: one distal to the first and another for closing the bottom of the mesial vertical release. 3-0 black silk suture in 19-mm reverse cutting needle was used for suturing.

After the surgery, both groups received verbal and written postoperative instructions. 500 mg amoxicillin capsule every 8 hours, 400 mg ibuprofen tablet three times a day, and daily 1.2% chlorhexidine mouthwash were prescribed for one week.

## Results

Soft tissue dehiscence rates, distal to the mandibular second molar after two flap designs, are listed in Table 1.

**Table 1:** Soft tissue dehiscence occurrence distal to the mandibular second molar after surgery using two different flap designs

	**Dehiscence at 1** ^st^ ** week**	**Extra appointment**	**Dehiscence size**	
			**X > 5mm**	**X < 5mm**
**Envelop flap group**	52	12	35	17
**N=120**				
**TTF group**	23	5	15	8
N=120				
TTF: Triangular transposition flap.				
X: The largest diameter of the hole, distal to the second molar measured with a Vernier ruler.				

There was no cases of infection in either group, but there were three cases of dry socket in the envelope group and four in the TTF group. In the envelope group, the dehiscence rate was 43% during the first week. The dehiscence was large (diameter more than 5 mm) in 67% of cases. Extra appointment rate (appointments requested by the patient exclusively related to the problem of the hole distal to the second molar) was 10% in the envelope group.

In the TTF group, dehiscence in the first week was 19%. Large dehiscence was present in 65% of cases. Extra appointments were requested in 4.1% of cases.

## Discussion

Wisdom tooth removal is probably the most prevalent outpatient surgery carried out by oral and maxillofacial surgeons and general dental practitioners.^13^

**Figure 3.**
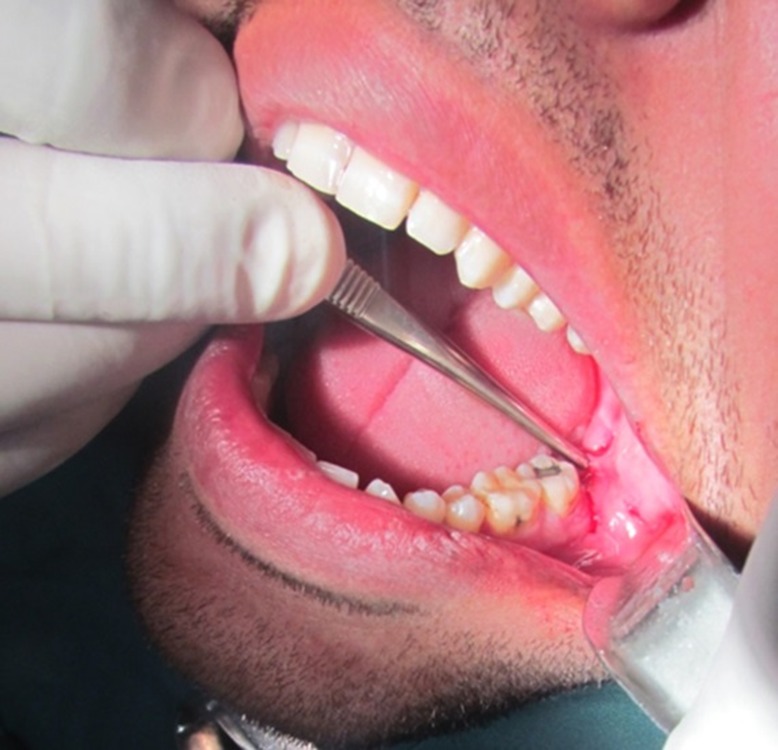
Triangular transposition flap (TTF) covers the wound created after surgical removal of wisdom tooth in the mandible and participative in primary wound healing.

Some postoperative complications after wisdom tooth removal, such as neurosensory changes in the lingual and mental nerve distribution, jaw fracture and tooth/root displacement are rare.^14-16^ However, symptoms like pain, swelling, trismus, infection, and bleeding are more common.^17^ Pain, swelling, and trismus are very common after wisdom tooth surgery. The majority of studies have focused on these routine post-operative complications.^18,19^ Despite its clinical significance, dehiscence after wisdom tooth removal has not been evaluated properly to date.^20-22^ Semi-impacted wisdom teeth often cause problems like caries, root resorption, and periodontal disease on the distal surface of mandibular second molar and infection and pericoronitis. Therefore, there is an increased request for their removal. Mesioangular impaction is also the most prevalent in the mandible.^23^ In the present study, dehiscence after surgical removal of mandibular class IB in both techniques was high; 43% and 19% in the envelope and triangular transposition flap groups, respectively. In both groups, the majority of the dehiscence cases had a large diameter. If soft tissue coverage is intact before removal of the wisdom teeth, the suturing is straightforward. Mucosal edges are brought together without tension. Even in this condition, there is a risk of wound dehiscence. In a study by Jakse et al,^24^ the incidence of wound dehiscence in 60 completely-covered mandibular third molars was 10% with the modified triangular flap design. In partially-erupted wisdom teeth, there will be a challenge in suturing after the operation: inadequate mucosa for tension-free approximation of buccal and lingual mucosa for primary closure of the surgical wound. Mucosal closure under tension will lead to wound dehiscence during the postoperative period. Other factors that can facilitate wound dehiscence include failure to remove the sulcular epithelium around the wisdom teeth and lack of bony support below the suture line. In the present study, no attempt was made to eliminate the sulcular epithelium during the operation. Triangular transposition flap (TTF) is a technique to tackle this problem. The transposed flap covers the extraction socket and suturing becomes tension-free (Figure 3). The envelope flap with wide elevation of the mucoperiosteum from the mandible reduces the tension during suturing.

This results of the this study showed that TTF can reduce dehiscence rate, more than the envelope flap, after wisdom tooth removal in mandibular mesioangular class IB impactions. TTF cannot completely prevent dehiscence due to the inherent nature of the surgical field with an unsupported soft tissue wound. Not all dehiscence cases will prompt the patient to refer to the dentist. A large dehiscence has more chance of being self-cleaning but in a small dehiscence, entrapment of food and bacterial fermentation products bother the patient. This explains why primary wound closure after removal of mandibular third molar leads to more pain and trismus in comparison with secondary healing, in which the surgeon intentionally creates 5-6 mm of gap in the mucosa distal to the mandibular second molar. The surgeons prefer to confront with an established large and self-cleaning dehiscence, rather than wound breakdown after closure of soft tissue flap under tension that might lead to small dehiscence. Tension-free primary wound closure without subsequent breakage can protect blood clot from lysis and prevent dry socket.^25^Otherwise, the risk of dry socket in this study was low and there was no significant statistical difference in this respect. A limitation of this study was the fact that extending the results of this study, carried out in a normal population, to patients using bisphosphonates cannot be reliable and further studies are necessary on the subject.

## Conclusion

The triangular transposition flap (TTF) may better prevent postoperative wound dehiscence in the surgical removal of mandibular third molar with mesioangular class IB impaction compared with the envelope flap.

## Acknowledgments

This study was supported by a grant from the Vice Chancellor of Research at Mashhad University of Medical Sciences. The results presented in this work have been taken from thesis No: 920850 registered at Mashhad University of Medical Sciences.
